# Effects of anesthetic agents on the evaluation of systolic and diastolic function in mice

**DOI:** 10.3389/fcvm.2025.1707937

**Published:** 2025-11-18

**Authors:** Dzmitry Matsiukevich, David M. Ornitz, Attila Kovacs

**Affiliations:** 1 Department of Developmental Biology, Washington University in St. Louis School of Medicine, St. Louis, MO, United States; 2 Department of Pediatrics, Washington University in St. Louis School of Medicine, St. Louis, MO, United States; 3 Department of Medicine, Washington University in St. Louis School of Medicine, St. Louis, MO, United States

**Keywords:** echocardiography, diastology, left atrium, cardiomyocyte, HFPEF, histology

## Abstract

**Aim:**

This study aimed to optimize non-invasive echocardiographic evaluation of myocardial function in a mouse model of diastolic heart failure, emphasizing the methodological challenges in assessing diastolic and left atrial (LA) function. Recognizing that clinical human studies frequently assess cardiac performance in non-sedated subjects, this investigation compared systolic and diastolic functional outcomes in mice subjected to heart failure with preserved ejection fraction (HFpEF) using two anesthetic agents: Avertin (mild sedation) and isoflurane (deep sedation). Additionally, we present a histological and echocardiographic correlation of the LA changes in established HFpEF mouse model.

**Results:**

Mice received angiotensin II and phenylephrine (AngII/PE) infusions for 28 days, followed by comprehensive echocardiographic and histologic analysis, including advanced diastology and LA assessment. AngII/PE treatment produced a reproducible HFpEF phenotype, with multiorgan involvement. Cardiac function measurements revealed significantly greater declines in both systolic and diastolic function in isoflurane-sedated mice, while mice sedated with Avertin primarily exhibited worsening diastolic metrics. LA histology corroborated imaging findings, showing profound wall thinning, reduced cellularity, and fibrotic conversion by day 28, changes tightly linked to deteriorating diastolic performance.

**Conclusion:**

The study highlights the limitations of deep sedation in accurately reflecting physiological cardiac function and underscores the importance of standardized mild-sedation protocols for translational murine heart failure research. Unlike the ventricular thickening and cardiomyocyte hypertrophy typically seen with diastolic dysfunction, LA remodeling was characterized by myocardial thinning and fibrosis, suggesting a distinct and opposite to LV pathogenic process. These findings support prioritizing minimally sedated echocardiographic assessment for better translational relevance.

## Introduction

Heart failure (HF) patients face a challenging prognosis, with statistics showing that only 50% survive beyond a five-year period post-diagnosis (1, 2). There are two fundamental types of heart failure; heart failure with reduced ejection fraction (HFrEF) which primarily affects systolic function, and heart failure with preserved ejection fraction (HFpEF), a syndrome that includes diastolic heart failure and multiorgan pathology. HFpEF affects ∼50% of patients with HF with no currently available cure. Correctly stratifying patients with HF is crucial, as recommended treatments target different mechanisms in HFrEF vs. HFpEF. Echocardiography (EC) is a well-established and essential non-invasive tool that has been used for the investigation of cardiac morphology and function. Conventional echocardiographic assessment includes two-dimensional EC that gives a visualization of the morphology of the heart; M-mode EC that allows assessment of systolic heart function, and Speckle Tracking Echocardiography (STE) and Tissue Doppler Imaging (TDI), a method of choice to measure blood flow or assess myocardial tissue velocity. Compared to conventional echocardiography, STE has emerged as an alternative to TDI and allows more precise evaluation of systolic and diastolic cardiac function including subclinical signatures of myocardial dysfunction (3). The structural remodeling of the LA is considered a hallmark of early diastolic dysfunction, serving a diagnostic role similar to “HbA1c in endocrinology” within practical diastology (4). In addition to assessing the LV and measuring circumferential, radial, and longitudinal strains along with LV rotational and torsional vectors, STE offers a valuable tool for evaluating LA performance. It is more effective than the left atrial volume index (LAVI) in determining the severity of HFpEF (5, 6). Furthermore, limitations of conventional echocardiographic parameters to reliably diagnose diastolic dysfunction and monitor different treatment conditions demands additional diagnostic tools including in depth assessment of the LA with STE analysis.

Clinical echocardiography is usually performed while the patient is awake and not exercising or with pharmacologically simulated exercise (stress echo). Cardiovascular effects of anesthesia includes but are not limited to hemodynamic change (changes in arterial and venous pressures, cardiac output, heart rate) as well as non-cardiac related adverse effects (temperature instability, respiratory depression, aspiration) (7). These undesirable sequalae may limit utilization of sedation, particularly in non-compliant pediatric population and in pre-existing compromised circulatory health states and may potentially result in cardiac arrest during the echo procedure. On the other hand, awake and stress echocardiography provides similar diagnostic and prognostic accuracy to advanced imaging modalities (8) improving translational value of the test while assessing cardiovascular reserves during physical activity and pathophysiological states requiring significantly increased oxygen delivery (e.g., sepsis).

It was shown that different depth of sedation (9) and anesthetic choice effects systolic ventricular function (10–12). Here, we compared inhaled anesthetic isoflurane with Tribromoethanol (Avertin) specifically focusing on LV and LA systolic and diastolic parameters. Consistent with previously known findings, mice anesthetized with isoflurane showed significantly depressed baseline systolic function compared to those with milder sedation using Avertin (10). In our established AngII/PE model of HFpEF, we observed significant worsening of conventional measures of diastolic function (E/E’ ratio, Tei index, and isovolumetric relaxation and contraction times (IVRT, IVCT). Additionally, non-conventional criteria such as left atrial ejection fraction (LA EF), left atrial strain (LA SR), and left atrial deformational index (LA DI) also showed significant decline. We confirmed the degree of diastolic dysfunction by performing retrograde LV catheterization with LV end diastolic pressure (LVEDP) measurements. We also identified histological changes in the LA consistent with an inflammatory response at early stages, and LA wall thinning and fibrosis at later stages of AngII/PE treatment. We hypothesized that LA changes in HFpEF are an autonomous manifestation of the multiorgan syndrome involvement and not determined by the severity of underlying LV disease. Though baseline systolic and diastolic parameters in control mice were significantly different with two different types of anesthesia, the ability to corelate echocardiographic values with real life physiologic performance were better when using low to moderate sedation with Avertin. With isoflurane, the technical aspect of echocardiographic image acquisition, technical reproducibility of echo images (11) and ability to interpret echocardiographic values at lower mouse heart rate were better.

## Methods

### Mice

Mice were housed in a pathogen-free facility and handled in accordance with standard use protocols, animal welfare regulations, and the NIH's Guide for the Care and Use of Laboratory Animals. Mice were housed with a 12 h light/dark cycle in a temperature (22 ± 1C^o^)- and humidity (55 ± 5%)-controlled room. Mice were allowed free access to water and a standard laboratory mouse diet (PicoLab Rodent Diet 20, Cat. No. 007688). All protocols were approved by the Washington University Animal Studies Committee. All mice were maintained on a C57BL/6J; 129X1 mixed, or F1 hybrid genetic background. Both male and female mice were used in these studies with approximately equal distribution.

### Mouse model for heart failure with preserved ejection fraction

Eight to ten-week-old mice were implanted with an osmotic mini-pump (Alzet, 200 μl, #2004) to allow subcutaneous infusion of angiotensin II and phenylephrine (AngII/PE) for 28 days.Mice were randomly selected and assigned to different groups: control mice (either no pump implantation or implantation of a pump filled with saline), and mice implanted with an infusion pump with AngII/PE. *Minipump insertion technique*. The mouse was placed in a prone position on a warming pad. The back was exposed, and the left lower quadrant was shaved. Under aseptic technique, an incision was made in the skin, and a subcutaneous tunnel was created using blunt dissection. An osmotic minipump was inserted into the tunnel, with the infusion port oriented towards the mouse's head (opposite to the incision site) to allow subcutaneous infusion of AngII/PE for 28 days. The incision was sutured with silk 4-0 to close the incision, and the mouse was placed in a recovery cage. Osmotic minipumps were programmed to deliver angiotensin II (1.5 μg/g/day) and phenylephrine HCl (50 μg/g/day), consistent with previous studies (13–15). Loaded minipumps were primed in 0.9% NaCl for 24 h at 37⁰C prior to implantation. Diastolic dysfunction in AngII/PE-treated animals was assessed using cardiac catheterization (see the Hemodynamic Analysis section). Mice exhibiting a significantly elevated LVEDP (>15 mmHg, not shown) were selected for the studies.

### Echocardiography

Transthoracic echocardiography with conventional 2D and doppler imaging and speckle tracking analysis was performed using a VisualSonics 3100 high resolution *in vivo* imaging system (VisualSonics, Toronto, Canada) equipped with a 30 MHz linear-array transducer at 0, and 28 days as previously described (16). The same mouse underwent echocardiography under Avertin anesthesia, followed by echocardiography under isoflurane anesthesia after a one-day recovery period. The isoflurane group were placed on 2.5% isoflurane for induction and 1%–1.5% isoflurane for maintenance of sedation, achieving a HR of 400–450 bpm. Body core temperature was continuously measured with a rectal probe and controlled using a homeothermic blanket system to prevent anesthesia-induced hypothermia. Cardiac electrical activity was monitored by ECG (Bio Amplifier, FE136, AD Instruments Ltd., UK). Cardiac images in the group anesthetized with Avertin (100 mg/kg i.p. tribromoethanol) were captured using a handheld echo probe technique on unrestrained mice placed on a heating pad to maintain physiological temperature control. Note that this anesthetic dose is one third of the dose used by Pachon et al. (10) and induces relatively light anesthesia with minimal effect on heart rate which ranged between 600 and 650 BPM. Echocardiographic techniques and assessment of conventional and non-conventional parameters (STE analysis of LA size and function) was performed as described (16).

### Hemodynamic analysis

Under general anesthesia (isoflurane) and full ventilatory support, retrograde catheterization was performed with a 1 French high fidelity micromanometer pressure catheter (SciSense Advantage System, London, Ontario, Canada). Systolic and diastolic blood pressure (BP) were recorded at 28 days in the right carotid artery and analyzed with SciSense software. The catheter was then advanced into the LV and LV systolic and diastolic pressures were recorded and analyzed with SciSense software.

### Histological analysis

**Figure 1:**
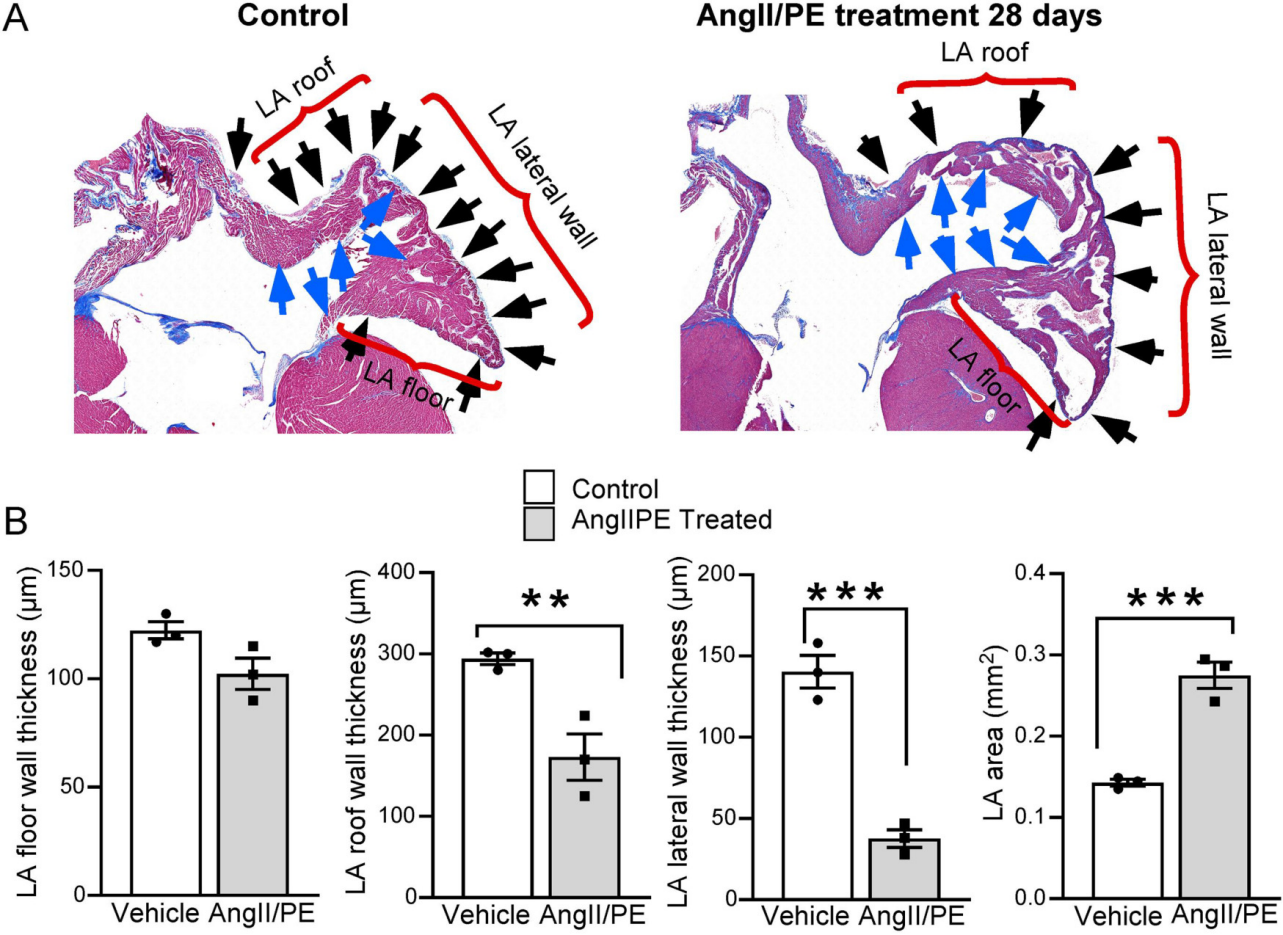
Quantitative analysis of LA histology. **(A)** Masson's Trichrome staining demonstrates LA wall thinning in AngII/PE treated compared to control mice. The LA is divided into three segments: the roof, the lateral wall, and the floor (brackets). Blue arrows demarcate the internal surface of the LA; black arrows indicate the external surface. **(B)** Quantitative analysis was performed separately on each of these segments. Results show a uniform thinning of the LA wall across all segments, with the most notable thinning observed in the floor of the LA. Additionally, treated animals demonstrated a significant enlargement in the overall LA area.

Heart samples were sectioned coronally to encompass all four chambers. Samples were collected with initial preservation in formalin, followed by a dehydration stage with 70% alcohol. Assessment of cardiomyocyte (CM) cross-sectional area was performed as previously described on 6 µm paraffin sections stained with wheat germ agglutinin (WGA) (17–19). A quantitative analysis was conducted to measure the myocardial wall thickness of the LA at three specific locations: the LA roof, LA lateral wall, and LA floor. The cross-sectional area of the LA was determined by measuring the intraluminal area at the level of the mitral valve within the endocardial boundary. The luminal portion of the LA was defined as the area internal to the endocardium (LA internal), as illustrated in Figure 1. ImageJ software was used for the analysis.

### Statistical analysis

Statistical analyses were performed using GraphPad Prism 9 software. Data are shown as mean ± SEM. For comparisons of multiple experimental groups, one-way ANOVA was used followed by *post hoc* Tukey's tests for pairwise comparisons. Student's *t*-tests were used for comparisons of two groups. Data with a *p* < *P* < 0.05 were considered statistically significant.

## Results

### Comparison of isoflurane and Avertin anesthesia on echocardiographic analysis

**Figure 2:**
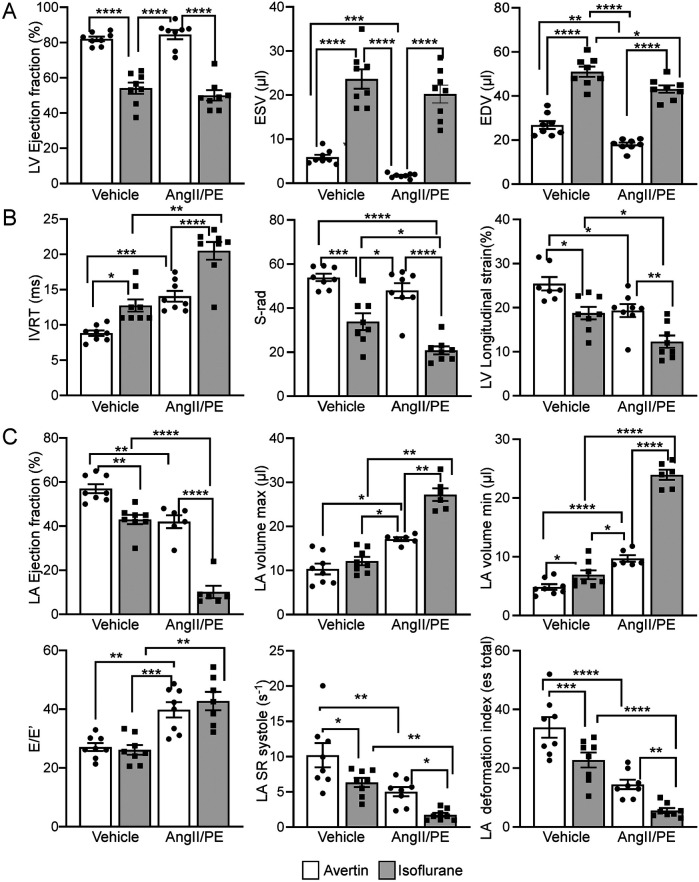
Comparison of isoflurane and Avertin anesthesia on echocardiographic systolic and diastolic parameters. **(A)** LV functional and volumetric echocardiographic parameters. **(B)** LV relaxation parameters **(C)** LA functional, volumetric and relaxation echocardiographic parameters.

Echocardiographic data and strain analysis reveal significant differences in cardiac function between mice anesthetized with Isoflurane vs. Avertin. Isoflurane anesthesia demonstrated a significant cardio depressive effect in control and AngII/PE treated animals in comparison to animals anesthetized with Avertin, where LV metrics showed marked reductions in LV EF and corresponding increased ventricular volumetric parameters (ESV and EDV) (Figure 2A, Table 1 and Supplementary Video 1A). No significant differences in systolic function parameters were observed within each anesthesia modality when comparing Isoflurane control to AngII/PE, and Avertin control to AngII/PE (Figure 2A and Table 1).

**Table 1:** Echocardiographic data for mice anesthetized with isoflurane vs. Avertin.

Left Ventricle												
Treatment and sedation modality	EDV	ESV	EF	SV	CO	dV/dt-s	dV/dt-s/EDV	dV/dt-s/InstV	dV/dt-d	dV/dt-d/ESV	dV/dt-d/instV	LVMdI
NaCl 28 days Avertin	26.8	5.9	82.7	20	13.1	0.77	28	42.3	0.74	125	39.3	2.8
NaCl 28 days Isoflurane	51.4^4*^	25.5^4*^	57.7^4*^	28.7^3*^	10*	0.79	15.3^4*^	41.1*	0.85	41^4*^	34	2.7
AngII/PE 28 days Avertin	18.8^#^	1.7^2#^	84.8	16.7^#^	9^3#^	0.76	40.4^3#^	42	0.57^2#^	362^4#^	29^#^	4.1^2#^
AngII/PE 28 days Isoflurane	44.5^4*1*#^	20^4*2#^	58.8^4*^	21.5^3*1#^	8^2#^	0.62^#^	13.5^4*^	21.3^3*2#^	0.83^2*^	39^4*^	24.7^#^	3.6^2#^

Left Ventricle Strain Analysis						
Treatment and sedation modality	S-rad	SR-rad-s	SR-rad-d	S-long	SR-long-s	SR-long-d
NaCl 28 days Avertin	53.3	15.3	14.6	25.8	15	13.9
NaCl 28 days Isoflurane	33.7^3*^	8.3^4^*	9.4^3*^	18*	6.9^4*^	7.1^4*^
AngII/PE 28 days Avertin	45^#^	13.6	13.3	19^#^	8.6^4#^	8.9^2#^
AngII/PE 28 days Isoflurane	20^4*1#^	7.9^4*^	8.1^3*^	12.3^2*1#^	4^2*1#^	4.4^2*1#^

Left Atrium Spectral Doppler Analysis															
			MV Annulus									Pulmonary veins			
Treatment and sedation modality	HR	E	IVCT	ET	IVRT	Tei Index	S’	E'	A'	E’/A’	E/E'	Peak S	Peak D	Mean S	Mean D
NaCl 28 days Avertin	595	770	5.6	38.7	9.1	0.34	37.8	29.9	24.1	1.26	25.8	171.5	662	121	404
NaCl 28 days Isoflurane	470^2^*	795	12.5*	44.3^2*^	12.8*	0.55*	31.5*	29	18.6*	1.45	26.2	220	470^3*^	131	289^4*^
AngII/PE 28 days Avertin	573	800	4.5	39^2#^	14.2^3#^	0.96^4#^	38.1	19^2#*^	26.1	0.78^#^	39.8^3#^	194	455^4#^	131	298^3*^
AngII/PE 28 days Isoflurane	430^2^*	999^#*^	22.5^4#3*^	45^#*^	20.6^4#*^	0.97^4#^	24.4^4#2*^	28	6.5^4#*^	1.8^3*^	42.7^3#^	102^3*4#^	513	69^3*3#^	307

Left Atrium Strain Analysis							
	LA Volumetric Indexes			LA Deformation Indexes			
Treatment and sedation modality	LA vol Max	LA vol Min	LA EF	*ε*s (total)	SR-s	SR-e	SR-a
NaCl 28 days Avertin	9.8	4.8	57	33.8	10.2	12.9	13.3
NaCl 28 days Isoflurane	12.1	6.9*	43^2*^	22.7^3^*	6.2*	5.9^4*^	5.9^4*^
AngII/PE 28 days Avertin	17.1^2#^	9.7^4#^	42^2#^	14.4^4#^	5^2#^	5.9^4*^	7.1^4#^
AngII/PE 28 days Isoflurane	27.2^2*2#^	23.9^4*4#^	10.2^4*4#^	5.6^2^*^4#^	1.7*^2#^	2.1^2^*^2#^	1.4^2*3#^

^*^p < 0.05, ^2^*p < 0.01; ^3^*p < 0.001; ^4^*p < 0.0001 - modality of treatment matched animals (comparison between controls with different anesthetics and AngII/PE treated animals with different anesthetics); ^#^*p* < 0.05, ^2#^*p* < 0.01; ^3#^*p* < 0.001; ^4#^*p* < 0.0001 - modality of anesthesia matched animals (comparison between the same anesthetic (avertin or isoflurane) and different treatment modality (AngII/PE vs. Control). *N* = 4 for each group (treated and untreated) and each sex (males and females).

HR, heart rate (BPM); EDV, end-diastolic LV volume (microliter); ESV, end-systolic LV volume (microliter); EF, ejection fraction (%); SV, stroke volume (microliter); CO, cardiac output (microliter/min); dV/dt, rate of change of LV volume; LVMI, LV mass index (mg/g); S-rad, average peak radial strain (%); SR-rad-s, average peak systolic radial strain rate (1/s); SR-rad-d, average peak diastolic radial strain rate (1/s); S-long, average peak longitudinal strain (%); SR-long-s, average peak systolic longitudinal strain rate (1/s); SR-long-d, average peak diastolic longitudinal strain rate (1/s); E, early mitral peak inflow velocity (mm/s); IVCT, isovolumic contraction time (ms); ET, ejection time (ms); IVRT, isovolumic relaxation time(ms); Tei Index, cardiac performance index (IVCT + IVRT/ET); S’, peak systolic velocity of mitral annulus (mm/s); E’, early diastolic velocity of mitral annulus (mm/s); A’, late diastolic velocity of mitral annulus (mm/s); E/E’, ratio of early mitral peak inflow velocity to early diastolic velocity of mitral annulus; PV Peak S, pulmonary vein peak systolic velocity (mm/s); PV Peak D, pulmonary vein peak diastolic velocity (mm/s); PV Mean S, pulmonary vein mean systolic velocity (mm/s); PV Mean D, pulmonary vein mean diastolic velocity (mm/s); LA vol max, maximal left atrial volume (microliter); LA vol min, minimal left atrial volume (microliter); LA EF, left atrial ejection fraction (%); εs (total), peak positive longitudinal LA strain corresponds to atrial reservoir function (%); SR-s, LA strain rate during ventricular systole corresponds to atrial reservoir function (1/s); SR-e, LA strain rate during early ventricular diastole corresponds to atrial conduit function (1/s).

The diastolic echocardiographic metrics revealed a significant decline in ventricular relaxation (IVRT, S-rad, LV longitudinal strain) in the Isoflurane control group, which continued to worsen in AngII/PE mice (Figure 2B and Table 1). The LA analysis highlighted considerable diastolic dysfunction in the Isoflurane control group, with reduced LA EF, increased LA volumetric parameters, elevated E'/E ratio, decreased LA strain (LA SR systolic, LA deformation index), with even more severe diastolic function parameters observed in AngII/PE treated animals (Figure 2C, Table 1 and Supplementary Video 1B).

Comparative analysis between male (*N* = 4) and female (*N* = 4) mice did not reveal any echocardiographic sex differences in either control or AngII/PE treated mice (Table 2).

**Table 2A:** Sex distribution of echocardiographic data (control group).

Left Ventricle												
Sex and sedation modality	EDV	ESV	EF	SV	CO	dV/dt-s	dV/dt-s/EDV	dV/dt-s/InstV	dV/dt-d	dV/dt-d/ESV	dV/dt-d/instV	LVMdI
♀ Nacl, Avertin	27.1	5.6	81.9	20.7	13.6	0.74	27.1	42.3	0.77	116	39.7	2.7
♂ Nacl, Avertin	26.6	6.2	82.2	19.4	13.8	0.8	29.5	41.8	0.72	134	39.6	3
♀ Nacl, Isoflurane	51.4^2#^	20.9^3#^	58.3^2#^	30.1^2#^	11.5	0.78	15.8^#^	45.7	0.84	38.6^#^	35.3	2.7
♂ Nacl, Isoflurane	50.6^2#^	26.3^3#^	49.9^4#^	27.1^#^	11.8	0.8	15.5^#^	36.9	0.77	43.5^#^	34.2	2.8

Left Ventricle Strain Analysis						
Sex and sedation modality	S-rad	SR-rad-s	SR-rad-d	S-long	SR-long-s	SR-long-d
♀ Nacl, Avertin	56	15.1	15.1	25.5	14.7	14.7
♂ Nacl, Avertin	51.7	15.6	14.4	25.1	15.2	13.1
♀ Nacl, Isoflurane	30.9^#^	9^#^	9.1^2#^	20^#^	6.8^2#^	7.8^2#^
♂ Nacl, Isoflurane	35.8^#^	7.6^2#^	9.7^#^	17^#^	6.9^2#^	7.7^2#^

Left Atrium Spectral Doppler Analysis															
			MV Annulus									Pulmonary veins			
Sex and sedation modality	HR	E	IVCT	ET	IVRT	Tei Index	S’	E'	A'	E’/A’	E/E'	Peak S	Peak D	Mean S	Mean D
♀ Nacl, Avertin	617	812	6.1	39.1	8.8	0.34	37.9	28.9	24	1.29	26.2	174	663	114	397
♂ Nacl, Avertin	601	763	5.2	39.8	8.8	0.34	37.7	30.8	23.8	1.23	28	169	614	129	410
♀ Nacl, Isoflurane	455^#^	818	12.8^2#^	45.9^#^	13^#^	0.5^#^	31^#^	29.3	19.1^#^	1.49	26.7	215	468^#^	139	306^#^
♂ Nacl, Isoflurane	459^#^	765	11.2^#^	46.5^#^	12.5^#^	0.5^#^	32	31.1	18.1^#^	1.4	24.9	225	473^#^	123	271^#^

Left Atrium Strain Analysis							
	LA Volumetric Indexes			LA Deformation Indexes			
Sex and sedation modality	LA vol Max	LA vol Min	LA EF	εs (total)	SR-s	SR-e	SR-a
♀ Nacl, Avertin	10.2	4.6	56	34.6*	9.9	13.3	13.7
♂ Nacl, Avertin	10.4	5	57	31.9*	10.5	12.7	12.9
♀ Nacl, Isoflurane	11.8	6.9	42.8^#^	25.3	6.7	5.7^#^	6.3^#^
♂ Nacl, Isoflurane	12.4	6.9	45.2^#^	20.3^#^	5.9	6^#^	5.6^#^

**Table 2B:** Sex distribution of echocardiographic data (AngII/PE treated group).

Left Ventricle												
Sex and sedation modality	EDV	ESV	EF	SV	CO	dV/dt-s	dV/dt-s/EDV	dV/dt-s/InstV	dV/dt-d	dV/dt-d/ESV	dV/dt-d/instV	LVMdI
♀ AngII/PE 28 days, Avertin	18.5	1.35	83.1	15.8	8.8	0.81	41.4	39.1	0.61	335	32.1	3.9
♂ AngII/PE 28 days, Avertin	17.5	1.65	85.2	17.5	9	0.69	39.3	44.2	0.54	369	29.7	4.1
♀ AngII/PE 28 days, Isoflurane	43.7^4#^	21.3^3#^	51.2^2#^	21.1^#^	8.7	0.66	12.8^3#^	21.6	0.86	24^2#^	26.1	3.6
♂ AngII/PE 28 days, Isoflurane	42.6^4#^	19.3^3#^	48.7^2#^	21.3^#^	7.6	0.58	13.9^3#^	27.1	0.81	41^2#^	23.2	3.8

Left Ventricle Strain Analysis						
Sex and sedation modality	S-rad	SR-rad-s	SR-rad-d	S-long	SR-long-s	SR-long-d
♀ AngII/PE 28 days, Avertin	50	14	14.6	19.1	9	10.1
♂ AngII/PE 28 days, Avertin	45.7	13.8	12.5	19.5	8.1	8.4
♀ AngII/PE 28 days, Isoflurane	24.1^2#^	9^#^	8.1^#^	11.9^#^	4.25^#^	5.5^#^
♂ AngII/PE 28 days, Isoflurane	17.5^3#^	7^#^	7.9^#^	12.6^#^	3.9^#^	3.6^#^

Left Atrium Spectral Doppler Analysis															
			MV Annulus									Pulmonary veins			
Sex and sedation modality	HR	E	IVCT	ET	IVRT	Tei Index	S’	E'	A'	E’/A’	E/E'	Peak S	Peak D	Mean S	Mean D
♀ AngII/PE 28 days, Avertin	573	823	3.8	39	12.8	0.98	39.7	19.5	27.5	0.75	41	197	443	129	283
♂ AngII/PE 28 days, Avertin	559	778	5.2	39.7	15.2	0.95	36.5	19.9	24.6	0.83	38	192	468	134	312
♀ AngII/PE 28 days, Isoflurane	460^#^	1,070^#^	20.1^2#^	46.7^#^	20^#^	0.98	24.5^2#^	29.7^#^	7^3#^	1.86^#^	43.5	100^#^	518	69.8^#^	299
♂ AngII/PE 28 days, Isoflurane	455^#^	948^#^	24.8^2#^	44.8^#^	21^#^	0.95	24.3^#^	27.9^#^	6^3#^	1.79^#^	42	104^#^	507	69^#^	315

Left Atrium Strain Analysis							
	LA Volumetric Indexes			LA Deformation Indexes			
Sex and sedation modality	LA vol Max	LA vol Min	LA EF	εs (total)	SR-s	SR-e	SR-a
♀ AngII/PE 28 days, Avertin	17	9	45.4	14.3	5.4	5.7	7.7
♂ AngII/PE 28 days, Avertin	16.9	10.5	46.3	14.5	4.5	6	6.6
♀ AngII/PE 28 days, Isoflurane	25.6^#^	22.7^4#^	10.9^3#^	5.6^2#^	1.8^2#^	2.4^2#^	1.6^#^
♂ AngII/PE 28 days, Isoflurane	28.5^#^	24.5^4#^	10.6^3#^	5.5^2#^	1.6^2#^	1.7^2#^	1.3^#^

# *p* < 0.05, ^2#*p*^ < 0.01; ^3#^*p* < 0.001; ^4#^*p* < 0.0001 - modality of sex matched animals (comparison between the same sex and different anesthesia: male avertin/isoflurane; female avertin/isoflurane); *p < 0.05, ^2*^p < 0.01; ^3*^p < 0.001; ^4*^p < 0.0001 - modality of anesthesia matched animals (comparison between the same anesthetic and different sex: avertin male/female; isoflurane male/female). *N* = 4 for each group (treated and untreated) and each sex (males and females).

HR, heart rate (BPM); EDV, end-diastolic LV volume (microliter); ESV, end-systolic LV volume (microliter); EF, ejection fraction (%); SV, stroke volume (microliter); CO, cardiac output (microliter/min); dV/dt, rate of change of LV volume; LVMI, LV mass index (mg/g); S-rad, average peak radial strain (%); SR-rad-s, average peak systolic radial strain rate (1/s); SR-rad-d, average peak diastolic radial strain rate (1/s); S-long, average peak longitudinal strain (%); SR-long-s, average peak systolic longitudinal strain rate (1/s); SR-long-d, average peak diastolic longitudinal strain rate (1/s); E, early mitral peak inflow velocity (mm/s); IVCT, isovolumic contraction time (ms); ET, ejection time (ms); IVRT, isovolumic relaxation time(ms); Tei Index, cardiac performance index (IVCT + IVRT/ET); S’, peak systolic velocity of mitral annulus (mm/s); E’, early diastolic velocity of mitral annulus (mm/s); A’, late diastolic velocity of mitral annulus (mm/s); E/E’, ratio of early mitral peak inflow velocity to early diastolic velocity of mitral annulus; PV Peak S, pulmonary vein peak systolic velocity (mm/s); PV Peak D, pulmonary vein peak diastolic velocity (mm/s); PV Mean S, pulmonary vein mean systolic velocity (mm/s); PV Mean D, pulmonary vein mean diastolic velocity (mm/s); LA vol max, maximal left atrial volume (microliter); LA vol min, minimal left atrial volume (microliter); LA EF: left atrial ejection fraction (%); εs (total): peak positive longitudinal LA strain corresponds to atrial reservoir function (%); SR-s, LA strain rate during ventricular systole corresponds to atrial reservoir function (1/s); SR-e, LA strain rate during early ventricular diastole corresponds to atrial conduit function (1/s).

### Histological analysis or the LA and LV

**Figure 3:**
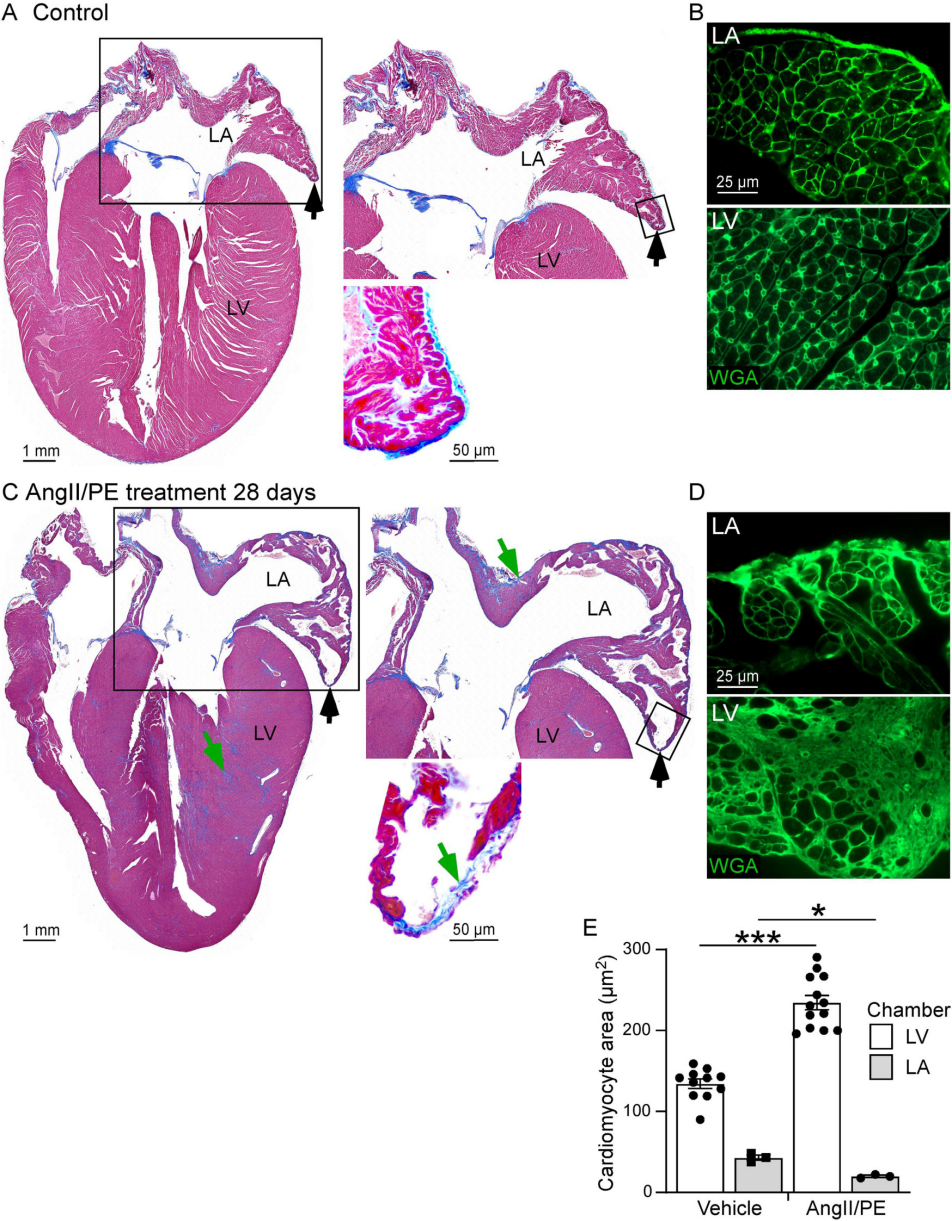
Histological analysis of the LA and LV. **(A,C)** Masson's trichrome stain showing area of EC deposition (blue, green arrows) and areas of LA wall thinning (black arrows) in control **(A)** and AngII/PE treated **(C)** wild type mice. (Right) High magnification of the LA area (box areas). **(B,D)** WGA staining (green, small-boxed region in A, C) demonstrates thinning of the LA and smaller cardiomyocyte cross sectional area in AngII/PE treated mice compared to control mice. **(E)** Quantitative analysis of cardiomyocyte cross sectional areas in the LA and LV. Cardiomyocyte cross sectional area was increased in the LV and reduced in the LA in AngII/PE treated compared to control mice. LA, left atrium; LV, left ventricle.

Hearts from six HFpEF and ten control mice were used for histological analysis. Histological sections of the heart, showing a four-chamber view, were stained with Masson's trichrome to identify areas of matrix deposition (blue) (Figures 3A,C). Particular attention was given to visualization of the LA and its structural juxtaposition with the LV. Whole LV analysis at 28 days revealed the presence of connective tissue/fibrosis (7%–9% of tissue area). In addition to LV histological changes, we also performed morphological analysis of the LA. Interestingly, the wall of the LA demonstrated thinning (as opposed to hypertrophy seen in LV) at late stages (28 days) of disease progression (Figure 1). CM cross sectional area was measured in WGA-stained heart tissue as an estimate of myocardial remodeling at the cellular level (Figures 3B,D). At baseline in control untreated mice CM cross sectional area was significantly smaller in the LA myocardium in comparison with connective tissue/fibrosis heart tissue (Figure 3E). Notably, in AngII/PE treated animals, LV CMs remodeled and demonstrated a hypertrophic response, with higher cross-sectional area, while LA CMs significantly thinned, with decreased CM cross sectional area in comparison to control LA CMs. (Figure 3).

## Discussion

The present study investigated the impact of anesthetic agents on the assessment of systolic and diastolic functions of the LV and LA in mice. This study suggests that careful selection of anesthetics for use in mice should be based on the cardiac functions being investigated and the choice of analytic techniques used to evaluate these cardiac functions.

Non-invasive methods for assessing cardiac output (CO) are essential for preclinical studies, particularly for longitudinal monitoring. Techniques such as echocardiography and MRI enable real-time assessment of cardiac function without the need for invasive procedures, reducing animal stress and mortality risks while allowing repeated measurements over time. However, as demonstrated in this study and other research (20, 21), the interpretation of non-invasive cardiac function measurements can be significantly affected by the choice of anesthetic agents. The specific anesthetic agent used may introduce bias into the study, potentially leading to incorrect conclusions. Furthermore, prioritizing the comfort of the echocardiographer by performing echocardiography on more heavily sedated mice rather than mildly sedated ones may not accurately reflect the physiological daily mouse routine conditions and extrapolate it to humans. In particular situations, such as the delineation of structural cardiac anomalies (22), this approach might be suitable, but it may not be ideal for assessing cardiac function. Concerns that unstressed, non-sedated mice might overly stimulate their autonomic sympathetic system can be addressed by monitoring objective parameters such as heart rate, which typically ranges from 500 to 700 bpm in a normal state.

The present study investigated the impact of two commonly used anesthetic agents, Isoflurane and Avertin (tribromoethanol), on the systolic and diastolic functions of the LV and LA in an AngII/PE mouse model of HFpEF and diastolic myocardial dysfunction. Our findings highlight significant alterations in cardiac function including dysfunction in control and untreated mice that are induced by different anesthetic agents, underscoring the importance of carefully selecting anesthetics in both experimental and clinical settings. The effects of anesthetic agents on systolic function assessment has been well described in a study that compared three (21) vs. five anesthetic agents under various depths of sedation (10). However, the impact of anesthetic agents, the selection of specific agents, and the level of sedation on the evaluation of diastolic function has primarily relied on outdated echocardiographic criteria (20) and has not been comprehensively studied, and additionally, non-cardiac effects of the anesthetics should also be taken into account (23, 24). Finally, identifying an ideal anesthetic agent to assess both systolic and diastolic dysfunction is even more critical and needs further investigation.

### LV systolic function

Cardiac contractility is the inherent capacity of the myocardium to contract. It is influenced by several factors, including preload, afterload, and intrinsic myocardial properties. Key echocardiographic values used to assess systolic function include EF, FS, SV, Myocardial perfusion imaging (MPI), and the relatively newer measure, Global Longitudinal Strain (GLS), which assesses myocardial deformation and corelates well with conventional EF measurements. Isoflurane, a commonly used volatile anesthetic, may alter preload (through vasoplegic effect on systemic venous vasculature), afterload (through vasomotor effect on arterial vascular bed) and intrinsic myocardial properties by targeting electron transport chain and ATP production in CMs (25). Despite findings aligned with previous studies suggesting that isoflurane has cardioprotective properties by minimizing myocardial depression, this quality of isoflurane has not been compared to other non-volatile (intravenous) agents (26). In addition, identical MAC (Monitored Anesthesia Care) delivery of the volatile anesthetic agent is rarely achieved unless mouse/patient are intubated. On the other hand, mild to moderate sedation achieved with Avertin demonstrated preserved EF without significant bradycardia. This suggests that Avertin may have a more favorable hemodynamic profile compared to volatile anesthetics like Isoflurane, especially in terms of maintaining cardiac function and avoiding significant bradycardia.

### LV diastolic function

LV diastolic function refers to the heart's ability to relax and fill with blood during the diastolic phase of the cardiac cycle. Increased filling pressures and ventricular stiffness can ultimately lead to diastolic dysfunction, which is a hallmark of HFpEF. This process is modulated by a complex interplay between myocardial relaxation, ventricular compliance, and filling pressures. Key physiological processes include Isovolumetric Relaxation, Early Rapid Filling, Diastasis and Atrial Contraction. Echocardiography is a valuable tool for assessing LV diastolic function, utilizing various parameters and techniques, including conventional (Transmitral Doppler Flow, Mitral Annular Motion, Pulmonary Venous Flow, LA Volume Index, Deceleration Time of E Wave) and non-conventional methods such as LA strain analysis (27). In our settings, we observed significant changes in LV diastolic function under different anesthetic conditions. A study comparison between Avertin and isoflurane anesthesia revealed a notable contrast in diastolic relaxation profiles. Despite the critical need to assess diastolic function, there is no consensus in the scientific community regarding the influence of different anesthetic agents on diastolic function. The existing body of research is limited, and findings are often inconsistent or inconclusive. Some studies suggest that anesthetic agents such as isoflurane or Avertin might influence diastolic function through their effects on myocardial relaxation and calcium handling (28); however, these results are not universally accepted or replicated across different research settings. The potential mechanisms proposed explaining diastolic dysfunction include alterations in calcium reuptake (29–31) within CMs, which can prolong diastolic relaxation and increase ventricular stiffness (32). Numerous confounding factors make it challenging to draw definitive conclusions, including variations in dosage, duration of anesthesia, underlying cardiac conditions, and methodological differences in assessing diastolic function. Although diastolic function remains relatively preserved, the primary issue noted in the literature with Avertin is its suppression of calcium reuptake within CMs (33). This inhibition prolonged the diastolic relaxation phase and increased ventricular stiffness, possibly due to impaired sarcoplasmic/endoplasmic reticulum calcium ATPase (SERCA) function (34). The resultant effect was a more prolonged isovolumetric relaxation time and reduced early diastolic filling. However, isoflurane significantly impaired diastolic relaxation more than Avertin, indicated by reduced early diastolic filling velocities and prolongation of isovolumetric relaxation time. These findings underscore the importance of considering the differential impacts of anesthetic agents on diastolic function, especially during cardiovascular studies. This understanding is also critical for optimizing perioperative care and preventing diastolic heart failure during surgeries.

### LA function

The LA is important to maintain normal cardiac function especially in the context of diastolic function. The LA acts not only as a conduit for blood flow from the pulmonary veins to the LV, but also serves as a reservoir during ventricular systole, and provides a booster pump function during late ventricular diastole. Volatile anesthetic agents have been demonstrated to adversely affect LA performance in patients with diastolic dysfunction, making the LA's booster function increasingly crucial for maintaining adequate filling and overall function (35, 36). Accurate assessment of LA function is essential in diagnosing and managing diastolic dysfunction. Our study highlights the significance of incorporating both conventional and non-conventional echocardiographic techniques in assessing LA function. LA rather than LV echocardiographic assessment, particularly under mild to moderate sedation with Avertin anesthesia, offers superior insights for the prediction of diastolic dysfunction. This approach may lead to better diagnostic and therapeutic strategies, enhancing our understanding and management of cardiac conditions associated with diastolic dysfunction.

### Histological changes in diastolic dysfunction: LV vs. LA

Diastolic dysfunction in the LV is often accompanied by well-documented histological changes, including myocardial hypertrophy, and extracellular matrix alterations, both of which further compromise myocardial relaxation and compliance. These structural changes collectively impair the LV's ability to relax and fill properly during diastole. In contrast, the LA's association with diastolic dysfunction is not as well characterized and largely focused on LA chamber dilation. Our histological analysis revealed distinct changes in CMs size, showing a reduction in size within the LA and hypertrophy within the LV.LA wall thinning and LA fibrosis are likely contributors to the functional impairment of the LA. Ongoing efforts in our study t. to validate the histological assessment of the LA in relation to its function will elucidate and highlight the underlying impairments observed in the LA to bridge this gap, future research should focus on integrating comprehensive histological evaluations. Such studies would enable a better correlation between functional alterations and morphological changes, thereby enhancing our understanding of the pathophysiology of diastolic dysfunction.

### Limitations of diastology protocols and investigations

Standard diastology protocols, though useful, have inherent limitations. The interpretation of diastolic function often relies on surrogate endpoints like E/A ratios and tissue Doppler imaging, which are influenced by loading conditions and heart rate, factors that anesthetics can significantly alter. Additionally, the small size and rapid heart rate of mice pose technical challenges, making it necessary to exercise caution and complement findings with multiple indices to ensure robust conclusions. In our study, we demonstrated excellent reproducibility and inter-rater reliability by relying on non-conventional methods of LA diastolic evaluation while mice were under mild to moderate sedation.

### Clinical and experimental implications

From an experimental viewpoint, choosing the appropriate anesthetic agent is critical to avoid confounding effects on cardiac measurements that could impair interpretation of phenotypes. In clinical practice, understanding the differential impacts of anesthetics allows for better management of patients with pre-existing cardiac conditions where maintaining optimal LV and LA functions is paramount.

Many studies commonly use isoflurane anesthesia due to its favorable safety profile and minimal hemodynamic disturbances, as well as the ease of handling anesthetized mice compared to handling awake animals. However, it is important to note several critical considerations:

#### Cardiodepressive effects

Although isoflurane is less cardiodepressive compared to other anesthetics, it still exerts some influence on cardiac function.

#### Impact on vascular resistance

Isoflurane can alter vascular resistance, introducing potential variability in hemodynamic measurements.

#### Heart rate modulation

Isoflurane affects heart rate, which can skew data related to cardiac function. Researchers utilizing isoflurane should be mindful of these factors and take them into account when interpreting their findings. In our Core Mouse Phenotyping Lab, by comparing isoflurane and Avertin anesthetic agents, we emphasize transitioning towards using very mildly sedated mice despite the time investment required to learn how to perform echocardiography on them.

#### Immobility induced hypothermia

A significant difference between small animals like mice and humans is the need for temperature regulation (37, 38). Mice require constant movement to maintain their body temperature. This phenomenon is somewhat comparable to the situation with newborn or premature babies, who also require artificial temperature control to regulate their body heat. It is well known that hypothermia is a direct and independent factor contributing to worsening diastolic dysfunction, which in turn leads to worsening systolic dysfunction (39). This underscores the importance of managing the depth of sedation in small species, as deeper sedation can lead to reduced movement, lower body temperature, and subsequently depressed heart function.

## Conclusion

In summary, the choice of anesthetic agents and depth of anesthesia profoundly affects both systolic and diastolic LV and LA function in mice including untreated control mice. We believe that un-sedated or mildly sedated rather than deeply sedated echocardiographic analysis represents a more physiological approach as it assesses cardiac output under homeostatic conditions. Incorporating non-invasive methods for cardiac output assessment during awake or physical exercise protocols can further enhance the accuracy of cardiac function evaluations. Finally, including endpoint histological assessments could provide deeper insights into the structural correlates of the observed functional changes. These considerations are essential to optimizing experimental designs and accurately interpreting cardiac function studies.

Future investigations could focus on elucidating the molecular mechanisms underlying these functional changes, potentially exploring alternative anesthetic regimens or adjunctive therapies to mitigate adverse effects. Additionally, long-term studies could provide more insights into the chronic impacts of anesthetic exposure on cardiac function.

## Data Availability

The raw data supporting the conclusions of this article will be made available by the authors, without undue reservation.

## Supplementary material

Supplemental Video 1 1A Parasternal long axis.

Supplemental Video 2 1B Modified 2-chamber view.
